# Emerging roles of proximal tubular endocytosis in renal fibrosis

**DOI:** 10.3389/fcell.2023.1235716

**Published:** 2023-09-20

**Authors:** Min Chen, Xiangchen Gu

**Affiliations:** ^1^ Department of Nephrology, Yueyang Hospital of Integrated Traditional Chinese and Western Medicine, Shanghai University of Traditional Chinese Medicine, Shanghai, China; ^2^ Department of Nephrology, Ruijin Hospital, Shanghai Jiao Tong University School of Medicine, Shanghai, China; ^3^ Department of Medicine, Shanghai Hospital of Civil Aviation Administration of China, Shanghai, China

**Keywords:** endocytosis, renal fibrosis, megalin, cubulin, tubule epithelial cell

## Abstract

Endocytosis is a crucial component of many pathological conditions. The proximal tubules are responsible for reabsorbing the majority of filtered water and glucose, as well as all the proteins filtered through the glomerular barrier via endocytosis, indicating an essential role in kidney diseases. Genetic mutations or acquired insults could affect the proximal tubule endocytosis processes, by disturbing or overstressing the endolysosomal system and subsequently activating different pathways, orchestrating renal fibrosis. This paper will review recent studies on proximal tubular endocytosis affected by other diseases and factors. Endocytosis plays a vital role in the development of renal fibrosis, and renal fibrosis could also, in turn, affect tubular endocytosis.

## Introduction

Endocytosis is a process of internalizing extracellular material intimately connected to diverse cellular functions, including signal transduction, cytoskeleton structure, and transcriptional regulation (Ellinger and Pietschmann, 2016). Several endocytic routes exist in eukaryotic cells, mainly receptor-mediated endocytosis and fluid-phased endocytosis. Receptor-mediated endocytosis involves clathrin-mediated and clathrin-independent endocytosis. The major endocytic way to internalize many cargoes is clathrin-mediated endocytosis (Kaksonen and Roux, 2018; Briant et al., 2020). Although clathrin-mediated endocytosis occurs in several renal cell types, endocytosis can also occur by non-clathrin-coated vesicles, including caveolae that contain a coat protein, caveolin (Zhuang et al., 2011; Srivastav et al., 2019). In kidneys, different cell types involve different endocytic pathways with other cellular functions. Glomerular cells, including endothelial cells, mesangial cells, and podocytes, all have the function of endocytosis, whereas tubular epithelial cells and collecting duct cells also have the same function (Singhal et al., 2000; Prabakaran et al., 2012; Wang et al., 2014; Inoue and Ishibe, 2015; Peng et al., 2016; Martin et al., 2018; Moriyama et al., 2019). This review mainly focuses on the endocytosis of proximal tubular epithelial cells and its relationship with renal fibrosis.

The proximal tubules reabsorb most filtered water and glucose (Eshbach and Weisz, 2017). The S1 and S2 segments of the proximal tubules internalize all the proteins filtered through the glomerular filtration barrier, including albumin and low molecular weight (LMW) proteins (Polesel and Hall, 2019). These LMW proteins include hormones (PTH, insulin, EGF, leptin, thyroglobulin), vitamin carrier proteins (transcobalamin-vitamin B12, vitamin D-binding protein (DBP), retinol-binding protein, folate-binding protein), enzymes (cathepsin B, plasminogen, urokinase, lysozyme), lipoproteins, cell surface antigen components (β2-microglobulin), immunoglobulin light chains, as well as drugs and toxins (aminoglycosides, gentamicin) (van der Wijst et al., 2019). The megalin (encoded by the *LRP2* gene), cubulin (encoded by the *CUBN* gene), and amnionless (encoded by *AMN* gene) complex, expressed at the brush border of the tubular epithelial cells, are the primary multi-ligand receptors in the receptor-mediated endocytosis in tubular epithelial cells (De et al., 2014). These receptors are constitutively internalized in clathrin-coated pits (CCPs) into small apical endocytic endosomes (AEEs) that fuse with large apical vacuoles (AVs) (Shipman and Weisz, 2020). The soluble contents of vacuoles are delivered to lysosomes, whereas membrane receptors, megalin, and cubilin are recycled to the apical surface (Smith et al., 2019; Shipman and Weisz, 2020).

Currently, endocytosis has been regarded as an essential component of many pathological conditions (Marzolo and Farfan, 2011). Increasing studies have focused on the role of tubular endocytosis in kidney disease development and progression. It is noted that various genetic mutations and acquired insults can affect PT endolysosomal function, leading to different cellular pathway activations and triggering proinflammatory and profibrotic responses, resulting in renal fibrosis (Dizin et al., 2013).

## Dent Disease

Dent disease is a rare genetic proximal tubulopathy (Gianesello et al., 2021). Mutations in the *CLCN5* and *OCRL* genes are known to cause Dent disease. Hoopes et al. first classified Dent Disease into three types, mutations in the *CLCN5* gene as Dent Disease type 1, mutations in *OCRL* gene as Dent Disease type 2, and no mutations in either gene are categorized as Dent Disease type 3 (Hoopes et al., 2004).

Dent disease type 1 is a heterotypic X-linked disease caused by mutations in *CLCN5*, which encodes the electrogenic Cl^−^/H^+^ exchanger CLC-5, localized in early endosomes of the proximal tubule (PT) (Gianesello et al., 2020). Phenotypic features commonly include low molecular weight proteinuria (LMWP), hypercalciuria, focal global sclerosis, calcium nephrolithiasis, nephrocalcinosis, and renal failure (Gorvin et al., 2013; Blanchard et al., 2016; Anglani et al., 2019). Disruption of the mouse *Clcn5* gene causes proteinuria by strongly reducing apical proximal tubular endocytosis, and both receptor-mediated and fluid-phase endocytosis and endosomal acidification are affected (Piwon et al., 2000). Reduced expressions of megalin and cubilin were also observed in Dent disease models (Christensen et al., 2003; Shipman and Weisz, 2020). Weisz et al. also discovered that decreased proximal tubule receptor expression and tubular proteinuria in Dent disease are primarily mediated by delayed early endosome maturation as a result of defective acidification and decreased [Cl -] accumulation (Shipman et al., 2023).

Lowe syndrome/Dent disease type 2 is caused by mutations in the phosphatidylinositol 4,5-bisphosphate (PtdIns4,5P(2)) 5-phosphatase OCRL, characterized by congenital cataracts, central hypotonia, and renal proximal tubular dysfunction (Suchy et al., 1995; De Matteis et al., 2017). Studies have shown that OCRL is localized to various endocytic compartments, suggesting impairments in the endocytic pathway as a possible disease mechanism (Sharma et al., 2015). Vicinanza et al. showed that OCRL controls early endosome (EE) function via its 5-phosphatase activity (Vicinanza et al., 2011). The further study analyzed that the defective receptor-mediated endocytosis was due to a megalin misplacement, in which it localizes in the endosomes instead of the brush border of the proximal tubular cells (Festa et al., 2019). Depletion of OCRL also impairs the recycling of multiple classes of receptors, including the megalin (Vicinanza et al., 2011). Using proteomic and metabonomic studies, Vilasi et al. demonstrated that tubular reabsorption of plasma-derived proteins was similarly reduced in Dent’s disease and Lowe syndrome, indicating that it is indistinguishable to identify Lowe syndrome and Dent’s disease (Vilasi et al., 2007).

Furthermore, Norden et al. demonstrated that tubular epithelial cells could not reabsorb a wide variety of polypeptides, hormones (insulin, growth hormone, insulin-like growth factor-1), and chemokines (monocyte chemoattractant protein-1) due to endocytosis impairment (Norden et al., 2001). Meanwhile, gene set enrichment analysis revealed that kidney development, anion homeostasis, organic acid transport, extracellular matrix organization, and cell migration biological processes are most likely involved in Dent Disease pathophysiology (Duran et al., 2021). Interestingly, in the presence of a defect in endocytosis, considerable compensatory reabsorption occurs further along the PT, presumably to limit the loss of proteins and their cargo into the urine (Polesel et al., 2022). These may partially imply the pathogenesis of tubulointerstitial fibrosis and may be one of the underlying mechanism contributing to the progressive renal failure in Dent disease (van Berkel et al., 2017).

## Cystic fibrosis

Cystic fibrosis is an autosomal recessive disease. It is a potentially lethal multisystem disease resulting from the accumulation of thick mucus that obstructs the airways, pancreatic ducts, intestine, bile ducts, and genital tract (Rowe et al., 2005). The disease-causing gene CFTR encodes a chloride channel and is also expressed in endosomes of proximal tubule cells (Riordan et al., 1989). CFTR mutations cause a major trafficking defect of endocytosis in proximal tubule cells, resulting in lysosomal dysfunction, oxidative stress, and tubular dedifferentiation/proliferation (Devuyst and Luciani, 2015). Further studies indicated that CFTR expression was downregulated in the kidney tissues of UUO murine model and CKD patients. Dysfunction or downregulation of CFTR in renal epithelial cells leads to alteration of genes in volved in EMT and kidney fibrosis (Zhang et al., 2017). Taken together, CFTR may affect endocytotic pathways but also involve in other classical profibrotic pathways. These changes combined together may affect long-term renal function, triggering tubulointerstitial injury and chronic kidney disease (Devuyst and Luciani, 2015).

## Cystinosis

Cystinosis is an autosomal recessive disease caused by mutations in the *CTNS* gene that encodes the lysosomal protein cystinosin, leading to an accumulation of cystine in all organs (Wilmer et al., 2010). Cystinosis could cause loss of expression of megalin/cubulin and impairment in endocytosis in PTCs(Gaide Chevronnay et al., 2014). Late-stage cystinosis progressed to kidney failure characterized by PTC atrophy with interstitial and glomerular fibrosis (Cherqui and Courtoy, 2017). A recent study also demonstrated that genetic ablation of the megalin/LRP2 pathway in cystinotic kidneys could suppress cystine accumulation and crystal deposition, and preserve kidney function from progression into renal fibrosis, indicating endocytosis impairment is the main pathway affecting cystinotic kidney injuries (Janssens et al., 2019). Using CRISPR/Cas9 technology, Krohn et al. generated *Ctns−/−* rats characterized by progressive cystine accumulation in the kidney, proximal tubule dysfunction, tubulointerstitial fibrosis, and kidney failure. Further primary cultures of proximal tubules from *Ctns−/−* rat kidneys confirmed the critical mechanism of cystinosis involved renal fibrosis, including cystine overload, leading to reduced endocytic uptake, increased proliferation, and defective lysosomal dynamics and autophagy (Zhang et al., 2017). Luciani et al. published a series of papers on the mechanisms of cystinosis involving tubular dysfunction. They found that in cystinosis, lysosomal dysfunction impairs autophagy’s ability to clear damaged mitochondria, resulting in oxidative stress that stimulates phosphorylation of tight junction ZO-1 by Gα12/Src and triggers a signaling cascade involving ZO-1-associated Y-box factor ZONAB, leading to tubular cell proliferation and transport defects (Festa et al., 2018). They also observed that defective cystine mobilization from lysosomes could divert PT cells toward growth and proliferation and disrupt their functions. The the primary mechanism is the activation of regulator-Rag GTPase-dependent recruitment of mTORC1 (mechanistic target of rapamycin complex 1) (Berquez et al., 2023). Altogether, these studies provide insightful information on the mechanism of cystinosis-related renal fibrosis, indicating reduced endocytosis and lysosomal and autophagic defects, leading to renal fibrosis.

## Ciliopathies

Ciliopathies are a group of diseases with mutations in ciliary-associated proteins. Many of these mutations manifest as renal ciliopathies, characterized by kidney dysfunction resulting from aberrant or ciliary functions, including polycystic kidney disease, nephronophthisis, et al. (McConnachie et al., 2021). In ADPKD, Obermuller et al. observed that loss of chloride channel CIC-5 and the albumin receptor megalin led to proteinuria in ADPKD (cy/+) rats, and the impairment in endocytosis could further reduce the efficacy of certain gene therapy (Obermuller et al., 2001; Witzgall et al., 2002). Endocytosis was further characterized as one of the shared gene pathways in ADPKD by the intra-species combined analysis (Chatterjee et al., 2017). Endocytic uptake was evident in megalin-positive cysts but only in those that remained connected to the renal tubular system (Nielsen et al., 2021). Altogether, many factors contributed to ADPKD induced renal fibrosis, and endocytic impairment occurred in ADPKD, leading to proteinuria, which may further partially orchestrate the development of renal fibrosis in ADPKD disease.

## Light chain kidney disease

Most kidney disorders associated with myeloma are caused by the excess production of monoclonal light chains, and renal involvement is almost always accompanied by light chain proteinuria (Batuman, 2007; Heher et al., 2013). Regular light chains are filtered through the glomerulus, endocytosed by the proximal tubule cells through multiligand endocytic receptors megalin/cubilin, and degraded in lysosome (Sanders, 2011). When significant amounts of light chains are generated by multiple myeloma and filtered through the glomerulus, the proximal tubular endocytic process is overloaded. Cell stress responses that include lysosomal alterations, phosphorylation of MAPKs, nuclear transcription factors NF-kB, Toll-like receptors, and STAT1, leading to the production of proinflammatory cytokines including TNFα, MCP-1 and Il1 β, and profibrotic cytokines like TGF-β1 (Batuman, 2007; Luciani et al., 2016; Ying et al., 2019; Sirac et al., 2021). Intracellular H_2_O_2_ induced by endocytosis of monoclonal free light chains oxidizes and activates c-Src, promoting the release of MCP-1 (Basnayake et al., 2010). As a result, these proximal tubule alterations often progress to severe tubulointerstitial kidney disease, the most common type of kidney involvement responsible for end-stage renal failure seen in myeloma patients (Batuman, 2007). It has also been shown that silencing megalin and cubilin genes may inhibit the uptake of myeloma light chain, subsequently suppressing inflammation in PTECs, and ameliorating nephrotoxicity (Li et al., 2008). Overall, endocytic overload of the light chain is the starting point, followed by a series of intracellular pathways activation, including pro-inflammatory and profibrotic pathways. Endocytosis overload caused by excess light chain production is the leading key role in tubular injuries and renal fibrosis in light chain kidney disease.

## Obesity-related renal disease

Obesity is significantly associated with the progression and development of chronic kidney disease (Kovesdy et al., 2017). It is demonstrated that megalin-mediated endocytosis involves the uptake of lipid-toxic glomerular-filtered substances, affecting the function of the endosome/lysosome system and resulting in autophagy impairment, followed by the increased production of profibrotic and inflammatory mediators in PTECs, which activates interstitial fibrocytes/pericytes to induce renal fibrosis (Kuwahara et al., 2016). It is believed that megalin could be a therapeutic target for obesity-related CKD. In obesity-related renal disease, numerous factors contribute to the development and progression of CKD, including insulin resistance, lipotoxicity, dysregulation of adipocytokine secretion, contribution of different fat depots, etc., (Sandino et al., 2022). Endocytosis impairment in tubule may partially contribute to CKD progression.

## Anti-brush border antibody renal disease

Anti-brush border antibody (ABBA) renal disease is identified as a unique and likely under-reported cause of severe and progressive renal tubular injury. In ABBA, circulating autoantibodies to the tubular brush border protein LRP2/megalin deposit in the TBM are characterized by IgG- and LRP2-containing immune complexes (Larsen et al., 2018; Dvanajscak et al., 2020). The disease course rapidly progresses to end-stage renal disease (ESRD) partially through endocytic impairment (Campbell et al., 2020).

## DM/DN affected tubular endocytosis

Numerous studies have been conducted to determine how tubular endocytosis is affected by Diabetes and Diabetic nephropathy. Interestingly, in early to mid-T2DM/DN, several groups found increases in megalin expression and albumin endocytosis due to the activation of insulin intracellular signaling, including PI3K/AKT, PKB, and mTOR pathways in TECs(Brunskill et al., 1998; Caruso-Neves et al., 2006; Hosojima et al., 2009; Koral et al., 2014; Peruchetti et al., 2014; Coffey et al., 2015; Bryniarski et al., 2018). Oxidative stress also increases megalin expression in the early stage of DM(Kurosaki et al., 2018). Other studies found that high glucose concentrations (HG) inhibited megalin expression (Tojo et al., 2001; Zhou et al., 2011). The mechanism is mainly because glucose is diverted to the hexosamine biosynthetic pathway (HBP), increasing O-GlcNAcylation of several intracellular proteins, including PKB (Peruchetti et al., 2018). PKB O-GlcNAcylation decreases PKB activity, leading to decreases in megalin expression and inhibition of the albumin endocytosis (Peruchetti et al., 2018). Then, as DN advances and glomerular selectivity decreases, PTECs are exposed to compounds like albumin, advanced glycation end products, and fatty acids bound to albumin, usually absent from glomerular filtrate (Saito et al., 2005a; Saito et al., 2005b; Abbate et al., 2006; Birn and Christensen, 2006; Tesch, 2008; Reidy et al., 2014; Slyne et al., 2015; Kuwahara et al., 2016; De et al., 2017). Excessive megalin/cubilin-mediated endocytosis overwhelms lysosomal clearance in PTECs and activates apoptosis, NLRP3 inflammation, and potentially tubulointerstitial fibrosis (Christensen et al., 2012; Liu et al., 2015; Kuwahara et al., 2016; De et al., 2017). As widely recognized, abundant factors are involved in DKD; in summary, tubular endocytosis is also greatly affected by DN/DKD, further adding to DKD progression.

## High salt diet-induced tubular endosomal injuries

Hypertensive individuals are at a higher risk for developing chronic kidney disease (CKD) characterized by interstitial fibrosis (Pugh et al., 2019). Tubular dysfunction has been observed in hypertensive patients and animal models (Wang et al., 2009; Landgraf et al., 2011), indicating the critical role of the tubule in hypertension-induced CKD. Further study revealed that a high salt diet reduced receptor-mediated endocytosis and the expression of megalin, as compared to a regular salt diet, leading to increases in pro-inflammatory and profibrotic factors (Silva-Aguiar et al., 2018; Teixeira et al., 2020). In addition, *in vitro* study also showed that incubation of LLC-PK1 cells with higher NaCl concentration decreased both albumin endocytosis and megalin expression (Teixeira et al., 2020). The underlying mechanism is also related to the increases in O-GlcNAcylation (Silva-Aguiar et al., 2018). In this part, these studies suggested that rather than commonly regarded that the high salt diet could increase blood pressure and indirectly cause renal injuries, high salt could also directly affect tubular endocytosis, leading to tubular injuries, resulting in renal fibrosis.

## Tubular reabsorption of albumin

Albumin is filtered by the glomeruli and reabsorbed by the proximal tubular cells through receptor-mediated endocytosis (Fatah et al., 2018). Megalin, cubulin, and AMN are all important since deficiency of any of the three proteins was shown to induce albuminuria in patients (Zeng et al., 2017; Bedin et al., 2020). Internalization of albumin by endocytosis is followed by transcytosis or undergo catabolism via lysosomal degradation (Gorriz and Martinez-Castelao, 2012; Molitoris et al., 2022). Low doses of albumin are protective. However, an albumin overload decreases the megalin expression and increases albumin endocytosis; overstressing the endocytic system is injurious to renal proximal tubule cells (Caruso-Neves et al., 2006). Albumin overload activates the mTORC2/PKB pathway leading to albumin-induced apoptosis (Caruso-Neves et al., 2006). Endocytosis of albumin induces Matrix Metalloproteinase-9 (MMP9) by activating the ERK signaling pathway in RTECs(Chen et al., 2017). Albumin endocytosis of PTECs could also lead to the activation of MCP-1 expression (Wang et al., 1997) and accumulation of collagen I, III, and IV (Wohlfarth et al., 2003). Since proximal tubular cells exposed to albuminuria exhibit a proinflammatory and profibrotic response, changes in proximal tubule (PT) albumin endocytosis play an essential role in developing tubular interstitial injuries (Teixeira et al., 2019). However, recently, another study revealed that serum, but not its major protein component albumin, induced tubular injury and secretion of proinflammatory cytokines (Lidberg et al., 2022). Altogether, further studies are required to elucidate the role of albumin in tubular epithelial injuries.

## Renal fibrosis and proximal tubular endocytosis

Renal fibrosis is the final manifestation of every type of chronic kidney disease (CKD) (Zhong et al., 2017). The hallmark of renal fibrosis is characterized by increased deposition of extracellular matrix (ECM), resulting in kidney function loss, leading to end-stage renal failure (Liu, 2006).

### Profibrotic factors downregulate megalin expression and affects endocytosis

TGF-β1 (transforming growth factor-β) has been identified as a central mediator in renal fibrosis (Meng et al., 2015). Interestingly, TGF-β1 downregulates megalin protein expression and reduces albumin endocytosis (Gekle et al., 2003). Further study showed that transcription factors SMAD2/3, the primary downstream pathway of TGF-β1, decrease megalin mRNA expression by binding directly to SBEs in the human megalin (Meng et al., 2015; Cabezas et al., 2019). Angiotensin II, another crucial role of renal fibrosis (Saldanha da Silva et al., 2017), also decreased megalin expression (Mezzano et al., 2001; Hosojima et al., 2009), and the *in vivo* inhibition of the angiotensin receptor (AT1) with Losartan protected against the reduction of megalin observed in the kidneys of proteinuria animal model (Marzolo and Farfan, 2011). Megalin also plays an important role in rhabdomyolysis-induced AKI, and megalin interference and inhibition could ameliorate rhabdomyolysis-induced AKI (Matsushita et al., 2021).

### Decreases in Dab2 exacerbates renal fibrosis

Notch is involved in kidney development and kidney injuries (Sirin and Susztak, 2012). Tubular epithelial notch reactivation also contributes to epithelial injuries and fibrosis development (Sirin and Susztak, 2012). Schutte-Nutgen et al. performed genome-wide transcriptome analysis to identify that the top downregulated gene of Notch signaling is Disabled-2 (Dab2) (Schutte-Nutgen et al., 2019). Dab2 is a cytoplasmic adaptor protein that binds to the cytoplasmic tail of the multiligand endocytic receptor megalin (Nagai et al., 2005). *Dab2* is involved in endocytic regulation and albumin endocytosis (Koral and Erkan, 2012). Knockout of *Dab2* in mice disrupts formation of endocytic vesicles, increases the excretion of vitamin D-binding protein, and reduces resorption of megalin (Morris et al., 2002; Nagai et al., 2005). Qiu et al. demonstrated that genetic lowering of *Dab2* expression in kidney tubules protected mice from renal fibrosis (Qiu et al., 2018). These data suggest that *Dab2* plays a versatile role in the kidney and impacts renal fibrosis.

### Loss of manba affects endo-lysosomal system of TECs and enhanced fibrotic response

MANBA (beta-mannosidase) is a lysosomal enzyme mainly expressed in the lysosome of TECs. Gu et al. showed that *Manba* knockout mice indicated a defect in lysosome function, leading to changes in receptor-mediated endocytosis and fluid-phase endocytosis and blockage in autophagy, followed by activation of the inflammasome and an enhanced fibrotic response after toxic injuries (Gu et al., 2021).

### Rab7 is involved in renal fibrosis through multiple mechanisms

Moreover, *Rab7*, a late endosome-/lysosome-associated small GTPase and an essential member of the Rab family, play critical roles in the endocytic processes (Zhang et al., 2009). *Rab7* participates in multiple regulation mechanisms in endosomal sorting, lysosome biogenesis, and phagocytosis (Zhang et al., 2009). Xu et al. showed that, in the Rab7-knock-in mice, the degree of renal fibrosis was milder than in WT mice on the seventh day of the UUO model. However, it became more severe 14 days after UUO(Xu et al., 2020). Liu et al. demonstrated that upregulation of *Rab7* relieved epithelial-mesenchymal transition (EMT) and apoptosis in albumin-treated TECs (Liu et al., 2018). The study also shows that autophagy regulates MMP-2 activity in a RAB7-dependent manner (Liu et al., 2018). Another study showed that long-term albumin stimulation combined with overexpression of Rab7 could decrease MMP-2 activity, exacerbate renal tubular injury, and accelerate the development of Tubular interstitial fibrosis (Liu et al., 2021). These results indicated that *Rab7* is closely associated with renal fibrosis progression and development.

Taken together, for the last part, we reviewed the recent studies on the cytokines like TGFβ1 and Angiotensin II and genes such as *Dab2*, *Rab7*, and *Manba*, affecting tubular endocytosis, eventually causing renal fibrosis. All these data also indicated that tubular endocytosis plays a crucial role in renal fibrosis.

## Concluding remarks

**FIGURE 1:**
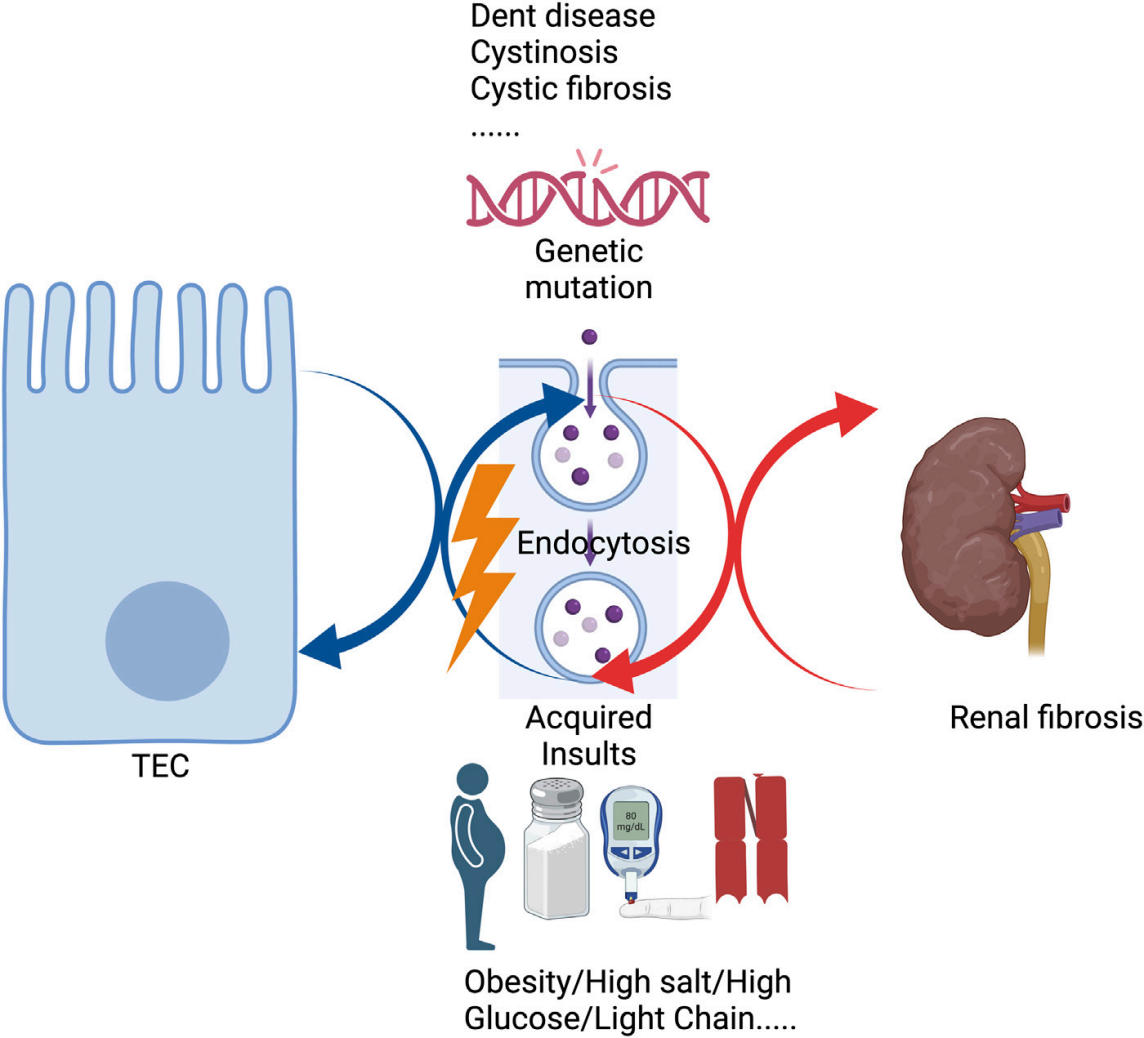
Genetic mutations or acquired insults affect tubular endocytosis, leading to renal fibrosis.

Overall, various genetic mutations and acquired insults, including high salt, high glucose, light chains, etc., could trigger tubular endocytosis injuries (Figure 1). As a result, tubular endocytosis impairment is either a causal factor or a contributing factor involved in renal fibrosis. Meanwhile, renal fibrosis exacerbates tubular endocytosis injuries in a feedback mechanism, leading to a vicious cycle (Figure 1). But current studies still lack precise mechanisms of the relationship between endocytosis and renal fibrosis, and further studies are needed to elucidate the specific role of endocytosis in renal fibrosis. This review also indicates that the endosomal pathway is an important therapeutic target for abrogating renal fibrosis, and megalin and several other genes may be a potential mechanism for drug targeting in renal fibrosis if initiated early in the disease.
